# Stress-induced translation of KCNB1 contributes to the enhanced synaptic transmission of the lateral habenula

**DOI:** 10.3389/fncel.2023.1278847

**Published:** 2023-12-13

**Authors:** Hakyun Ryu, Minseok Kim, Hoyong Park, Han Kyoung Choi, ChiHye Chung

**Affiliations:** ^1^Department of Biological Sciences, Konkuk University, Seoul, Republic of Korea; ^2^Department of Brain and Cognitive Sciences, Daegu Gyeongbuk Institute of Science and Technology (DGIST), Daegu, Republic of Korea

**Keywords:** lateral habenula, KCNB1, translating ribosome affinity purification (TRAP), synaptic transmission, depression, stress, bursting, ion channel

## Abstract

The lateral habenula (LHb) is a well-established brain region involved in depressive disorders. Synaptic transmission of the LHb neurons is known to be enhanced by stress exposure; however, little is known about genetic modulators within the LHb that respond to stress. Using recently developed molecular profiling methods by phosphorylated ribosome capture, we obtained transcriptome profiles of stress responsive LHb neurons during acute physical stress. Among such genes, we found that KCNB1 (Kv2.1 channel), a delayed rectifier and voltage-gated potassium channel, exhibited increased expression following acute stress exposure. To determine the roles of KCNB1 on LHb neurons during stress, we injected short hairpin RNA (shRNA) against the *kcnb1* gene to block its expression prior to stress exposure. We observed that the knockdown of KCNB1 altered the basal firing pattern of LHb neurons. Although KCNB1 blockade did not rescue despair-like behaviors in acute learned helplessness (aLH) animals, we found that KCNB1 knockdown prevented the enhancement of synaptic strength in LHb neuron after stress exposure. This study suggests that KCNB1 may contribute to shape stress responses by regulating basal firing patterns and neurotransmission intensity of LHb neurons.

## Introduction

Stress is a prevalent issue in contemporary society and a major risk factor for psychiatric disorders like major depressive disorder (Kendler et al., 1999; Yang et al., 2015). Stress certainly impacts neuronal and synaptic properties in various brain areas and one such area is the lateral habenula (LHb), a part of the epithalamus (Li et al., 2011; Proulx et al., 2014; Kim and Chung, 2021). Animal models of depression have demonstrated potentiated synaptic activities (Li et al., 2011; Park et al., 2017a) as well as increased neuronal activity in the LHb (Liu et al., 2021). An intriguing aspect of LHb neurons is their heterogeneity, as they can be divided into several subpopulations based on anatomical (Hu et al., 2020) or molecular differences (Hashikawa et al., 2020; Wallace et al., 2020). For instance, the intensity of stress may determine the number of activated neurons within the LHb but not all neurons in the LHb are stress-responsive (Park et al., 2017b). Circuit-specific studies focusing on specific projecting areas, such as the ventral tegmental area, dorsal raphe nucleus, and hypothalamus have provided well-established insights into the LHb’s involvement in animal models of depression (Li et al., 2011; Liu et al., 2021; Zheng et al., 2022). Additionally, increased expression of certain proteins (e.g., p11 and βCaMKII) has been shown to enhance LHb activity and mediate depression-like behaviors (Li et al., 2013; Seo et al., 2018), suggesting that molecular switches responding to stress may underlie the observed heterogeneity. However, little is known about the specific molecular modifications that occur in the LHb during stress exposure.

Therefore, taking advantage of the newly developed selective capturing technique of phosphorylated ribosomes (Knight et al., 2012), we aimed to obtain an unbiased molecular profile of the activated LHb during acute physical foot shock stress. This systemic approach revealed a list of genes of interest that are transcriptionally regulated during stress exposure, and among them, the *kcnb1*, which encodes KCNB1 (Kv2.1 channel), exhibited a significant increase during stress.

KCNB1 is a delayed-rectifier voltage-dependent potassium channel, located on the soma and proximal dendrites of neurons (Du et al., 2000), and expressed in LHb neurons (Mandikian et al., 2014). Similar to other voltage-gated potassium channels (Pongs, 1999), KCNB1 regulates action potential width and neuronal excitability (Malin and Nerbonne, 2002; Liu and Bean, 2014). Previously, it is reported that chronic stress increases the expression of KCNB1, while antidepressant treatment decreases their enhanced expression in stressed animals (Chen et al., 2015; Wang et al., 2020). In the LHb, extracellular potassium concentration may contribute to depressive phenotypes by increasing neural activity in depression animal models (Cui et al., 2018), and antidepressant treatment has been shown to block this enhanced neural activity in LHb neurons and alleviate depressive-like behaviors (Yang et al., 2018a). Based on these findings, we hypothesized that enhanced KCNB1 expression during acute stress contributes to the LHb activity and influences the response to stress. To test our hypothesis, we virally delivered short hairpin RNA (shRNA) against *kcnb1* genes to reduce the expression of these specific channels in the LHb and examined the impact of KCNB1 knockdown on basal synaptic transmission and behaviors.

## Materials and methods

### Animals

All experiments except for phosphor-TRAP used five- to nine-week-old C57BL/6 N mice, purchased from Orient Bio (Gapyeong, South Korea). The animals were group-housed (four mice per cage) with free access to food and water until the stereotactic surgery was performed. Following surgery, the animals were individually housed under controlled laboratory conditions, which included a 12-h light/dark cycle with lights on at 7 am, humidity maintained at 45–50%, and a temperature of 23 ± 2°C. All animal experiments were conducted in accordance with the guidelines set by the National Institutes of Health (NIH) for animal use and care. All the experiments were approved by the Institutional Animal Care and Use Committee (IACUC) of Konkuk University (KU22005, Seoul, South Korea).

For the phospho-TRAP experiment, eight- to ten-weeks-old C57BL/6 J mice were purchased from Hyochang Science (Daegu, south Korea). These animals were group-housed with four mice per cage and provided with *ad lib* access to food and water. All procedures involving these mice were approved by the IACUC of Daegu Gyeongbuk Institute of Science and Technology (DGIST).

### Translating phosphorylated ribosome affinity purification (phospho-TRAP)

Phospho-TRAP analysis was performed as in a previous report (Knight et al., 2012), with the following procedure. Protein A Dynabeads (Invitrogen) were pre-coupled with phospho-S6 antibody (Invitrogen #44-923G) in pre-coupling buffer (10 mM HEPES [pH 7.4], 150 mM KCl, 5 mM MgCl_2_, 1% NP40, and 0.05% IgG-free BSA) for overnight at 4°C (Proten A Dynabeads 1 mL with 2 ug antibody). Beads were washed two times with 0.15 M KCl Wash Buffer (10 mM HEPES [pH 7.4], 150 mM KCl, 10 mM MgCl_2_, and 1% NP40) immediately before use.

After either foot shock or sham handling, mice were sacrificed by cervical dislocation, followed by immediate decapitation. Brains were extracted and placed in chilled cooling buffer (1x PBS, 5 mM NaF, 2.5 mM Na_3_VO_4_, 2.5 mM Na_4_P_2_O_7_, 5 mM β-Glycerophosphate, 100 μg/mL cycloheximide, and Roche cOmplete protease inhibitor [1 tablet per 100 mL of the buffer]) to remove blood. Using the brain matrix, we dissected 1-mm thick coronal slices containing the LHb. The LHb was rapidly collected by 2-mm diameter punch while the brain slices were in the dissection buffer (1x HBSS, 4 mM NaHCO_3_, 2.5 mM HEPES [pH 7.4], 35 mM Glucose, and 100 μg/mL cycloheximide). The collected LHb tissue was transferred to a glass homogenizer (Kimble Kontes 20) and resuspended in 0.5 mL of homogenization buffer (10 mM HEPES [pH 7.4], 150 mM KCl, 10 mM MgCl_2_, 100 nM calyculin A, 0.5 mM DTT, 100 Units/ml RNasin, 250 μg/mL cycloheximide, and Roche cOmplete protease inhibitor [1 tablet per 10 mL of the buffer]). Samples were homogenized at 900 rpm for 2 min with 12 strokes on automated tissue homogenizer at 4°C. Homogenates were transferred to a microcentrifuge tube and clarified at 2,000 xg for 10 min at 4°C. The supernatant was transferred to a new tube, and 35 μL of 10% NP40 and 35 μL of 1,2-diheptanoyl-sn-glycero-3-phosphocholine (DHPC, Avanti Polar Lipids: 100 mg/ 0.69 mL). The solution was gently inverted to mix and incubated for 2 min on ice. The solution clarified at 20,000 xg for 10 min at 4°C. The supernatant was transferred to a new tube. A 50 μL aliquot of this solution was removed, transferred to a new tube containing 350 μL buffer RLT (QIAGEN) as total RNA sample, and the remainder was used for immunoprecipitation.

Immunoprecipitations were allowed to proceed overnight at 4°C. The beads were then washed four times with 0.35 M KCl Wash Buffer (10 mM HEPES [pH 7.4], 350 mM KCl, 10 mM MgCl_2_, 100 nM calyculin A. 0.5 mM DTT, 100 Units/ml RNasin, 1% NP40, and 100 μg/mL cycloheximide). During the third wash the beads were transferred to a new tube and allowed to incubate at RT for 10 min. After the final wash the RNA was eluted by addition of buffer RLT (350 μL) to the beads on ice, the beads removed by magnet, and the RNA purified using the RNeasy Mini Kit (QIAGEN). RNA assessed using an Agilent Fragment Analyzer system. For next generation sequencing, cDNA was synthesized using the Ovation RNA Amplification System V2 (NuGEN) and then sequenced using an Illumina HiSeq 2000. Quality control and analysis of sequenced data were performed by commercial service (SYSOFT, Daegu, South Korea) using FastQC (Wingett and Andrews, 2018) and HTseq (Anders et al., 2015).

### Tissue sampling and Western blot

For tissue sampling, brain slices were prepared in chilled sucrose dissection buffer using Leica VT1000s vibratome. The habenula complex or the LHb was specifically dissected using a sampling tool (WellTech, Rapid-core, 0.5 mm tip) under a microscope (Nikon) for phospho-TRAP or Western blot, respectively. The obtained tissues were stored in RIPA buffer (Cell Signaling) containing a protease inhibitor cocktail (Roche) and placed at −80°C. Western blotting was then performed as described previously (Park et al., 2017a). The antibodies used were anti-KCNB1 (1:1000, Neuromab, #75–014) and anti-GAPDH (1,5,000, Sigma-Aldrich, #G9545).

### Virus information

AAV1-shRNA-ctrl-EGFP virus was purchased from Addgene (Addgene plasmid # 85741; from Hongjun Song) (Yu et al., 2015). Scrambled RNA (scRNA) sequence (5’-CCTAAGGTTAAGTCGCCCTCG-3′) and shRNA sequence against *kcnb1*(5’-GCCTTGGAGCTAGAACAGAAATTCAAGAGATTTCTGTTCTAGCTCCAAGGCTTTTTT-3′) were purchased from the Institute for basic science (IBS) virus facility (Daejeon, Korea). The *kcnb1* shRNA cloning and viral packaging were also carried out by IBS.

### Surgery and virus injection

Male C57BL/6 N mice (4–5 weeks old) were deeply anesthetized with isoflurane for the stereotaxic surgery to deliver shRNA against the *kcnb1* gene. The AAV virus vector was bilaterally injected (0.3 μL each) into the LHb (in mm: AP -1.48, ML ±0.45, DV -3.0) using a blunt needle (33 gauge, NanoFil) connected to a 2 μL Hamilton syringe (25-gauge) with PE tubing (C313CT) to a syringe pump (Fusion 100, Chemyx) at a rate of 0.08 μL/min. After the surgery, Rimadyl (Carprofen) was diluted with saline (0.5 mg/mL) and subcutaneously injected (0.01 mL/g) to alleviate pain. The mice were then placed on a warm plate for 1 h to aid in recovery. All animals were single-housed, and experiments were carried out a minimum of 2 weeks after surgery.

### Stress paradigm

All subjects were placed in the foot-shock chamber (H10-11 M-TC, Collbourn Instruments, Whitehall, PA, United States) and received a total of 360 foot-shocks (0.3 mA intensity, 3 s duration, 4–10 s of inter-stimulus intervals) for three consecutive days to induce acute learned helplessness (aLH) in animals. Behavior tests and electrophysiological recording were conducted 24 h after the last shock session of aLH.

### Slice preparation

All mice (naïve and aLH uninfected, naïve and aLH scRNA, naïve and aLH shRNA) were anaesthetized with 100% isoflurane, decapitated, and their brains were immediately extracted and placed in chilled sucrose dissection buffer (containing in mM: 212 sucrose, 3 KCl, 26 NaHCO_3_, 1.25 NaH_2_PO_4_, 7 MgCl_2_, 10 glucose, bubbled with 95% O_2_ and 5% CO_2_). Using a Leica VT1000s vibratome, 300 μm thick coronal slices containing the LHb were prepared. Before recording, all brain slices were stored in a recovery chamber containing artificial cerebrospinal fluid (aCSF, containing in mM: 1 NaH_2_PO_4_, 26.2 NaHCO_3_, 118 NaCl, 2.5 KCl, 11 glucose, 2 CaCl_2_, 1 MgCl_2_ and bubbling with 95% O_2_ and 5% CO_2_) at a 35°C water bath for 50–60 min, and then transferred to room temperature.

### Electrophysiological recording

A brain slice was transferred to a recording chamber constantly perfused with aCSF at 29–31°C. Whole-cell patch-clamp recordings were conducted using Multiclamp 700B and Clampex 10.3 (Molecular Devices), sampled at 10 kHz, and filtered at 5 kHz. The recordings were collected only if they met our criteria, which required that holding currents be less than 100 pA and access resistance (R_a_) be lower than 25 MΩ. The group-specific passive properties (R_a_ and membrane resistance, R_m_) obtained from the recordings are presented in the table (Table 1). Glass recording pipettes with resistances of 3–6 MΩ were used. Resting membrane potentials (RMPs) and basal firing patterns were recorded at a holding current of 0 pA. Because RMP was different between groups, membrane potential was adjusted to – 60 mV to minimize influence of Vm on firing properties before recording. The threshold of firing was recorded by injecting a series of current steps (20 steps with a 15 pA increment from 0 pA, 15 ms duration), and excitability was recorded by injecting 500 ms long current steps (11 steps with a 20 pA increment from 0 pA). Miniature excitatory postsynaptic currents (mEPSCs) were recorded at a holding potential of - 60 mV with 1 μM tetrodotoxin and 50 μM picrotoxin in the aCSF from EGFP-expressing neurons exclusively in scRNA/shRNA injected group and random neurons in uninfected group. To block the KCNB1, we added specific KCNB1 channel blocker, GxTx-1E (100 nM), in the aCSF. Current-clamp recording was performed using K-gluconate based internal solution contained the following (in mM): 103 K-gluconate, 10 HEPES, 0.6 EGTA, 5 KCl, 2.5 Mg-ATP. Voltage-clamp recording was performed using Cs-internal solution (containing in mM: 115 Cs-methanesulphonate, 20 CsCl, 10 HEPES, 2.5, MgCl_2_, 0.6 EGTA, 5 QX314, 4 Na_2_-ATP, 0.4 Na_2_-GTP, and 10 Na-phosphocreatine) The ionic equilibrium potential for potassium (E_K_) was calculated by Nernst equation based on the concentration of K^+^ in internal solution and aCSF.

**Table 1:** Access resistance (R_a_) and membrane resistance (R_m_) value from current-clamp recordings of LHb neurons.

Parameters	*uninfected naïve*	*uninfected aLH*	*Naïve + shRNA*	*aLH + shRNA*
Access resistance (R_a_, MOhms)	10.77 ± 0.72	11.21 ± 0.77	14.40 ± 0.88^**^	15.19 ± 1.07^**^
Membrane resistance (R_m_, MOhms)	576.43 ± 89.21	774.12 ± 101.63	510.07 ± 52.03	631.40 ± 63.13

R_a_ was significantly increased by genetic modulation (Two-way ANOVA with LSD post-hoc analysis, main effects of gene modulation, F_1, 95_ = 18.246, *p* < 0.0001; main effects of stress exposure, F_1, 95_ = 0.484, *p* = 0.488; interaction, F_1, 95_ = 0.040, *p* = 0.842; LSD post-hoc analysis, *p* = 0.007 in comparison between Uninfected naïve and Naïve + shRNA; *p* = 0.001 in comparison between Uninfected aLH and aLH + shRNA), however, there were no significant differences in R_m_ (*n* = 20–27, Kruskal-Walis test, *p* = 0.065).

### Active avoidance task (AAT)

The AAT box consisted of two chambers that separated by the guillotine door and grid bottom that can deliver electric shocks (Avoidance system, version 1.1; B.S technolab Inc., Seoul, Korea). All subjects were placed in one camber of the AAT box and habituated for 5 min. Following the habituation, mice underwent a total of 30 trials stimuli, which included a conditioned stimulus (2 s, sound, 20% intensity, 5 kHz frequency) and an unconditioned stimulus (foot shock, 0.3 mA intensity, 10 s duration, 12–36 s of inter-stimulus intervals). Subjects had the option to escape the food shock by moving to the opposite chamber through the guillotine door. If mice passed through the door during shock delivery, it was considered an escape (success), whereas if mice remained in the chamber during shock, it was considered a failure. The number of failures and the latency to escape was measured to quantify the helplessness.

### Data analysis and statistics

Electrophysiological data were analyzed by Mini Analysis software (Synaptosoft) and Clampfit 10.4 (Molecular Devices). All presented data are mean ± SEM value. The relative distribution of firing pattern data was statistical analyzed by the Pearson chi square test. Other statistic values were analyzed by Student’s t tests, One-way ANOVA or Two-way ANOVA. Outlier data points were excluded from all data. Normality and homoscedasticity were tested by Shapiro–Wilk and Levene’s test, respectively. If data set violates the assumption of parametric statistics, Mann–Whitney or Kruskal-Walls tests were used. *p*-values less than 0.05 are considered statistically significant and the levels of significance are indicated by asterisks (* *p* < 0.05, ** *p* < 0.01, *** *p* < 0.001).

## Results

### KCNB1 is readily translated in the LHb during stress

**Figure 1:**
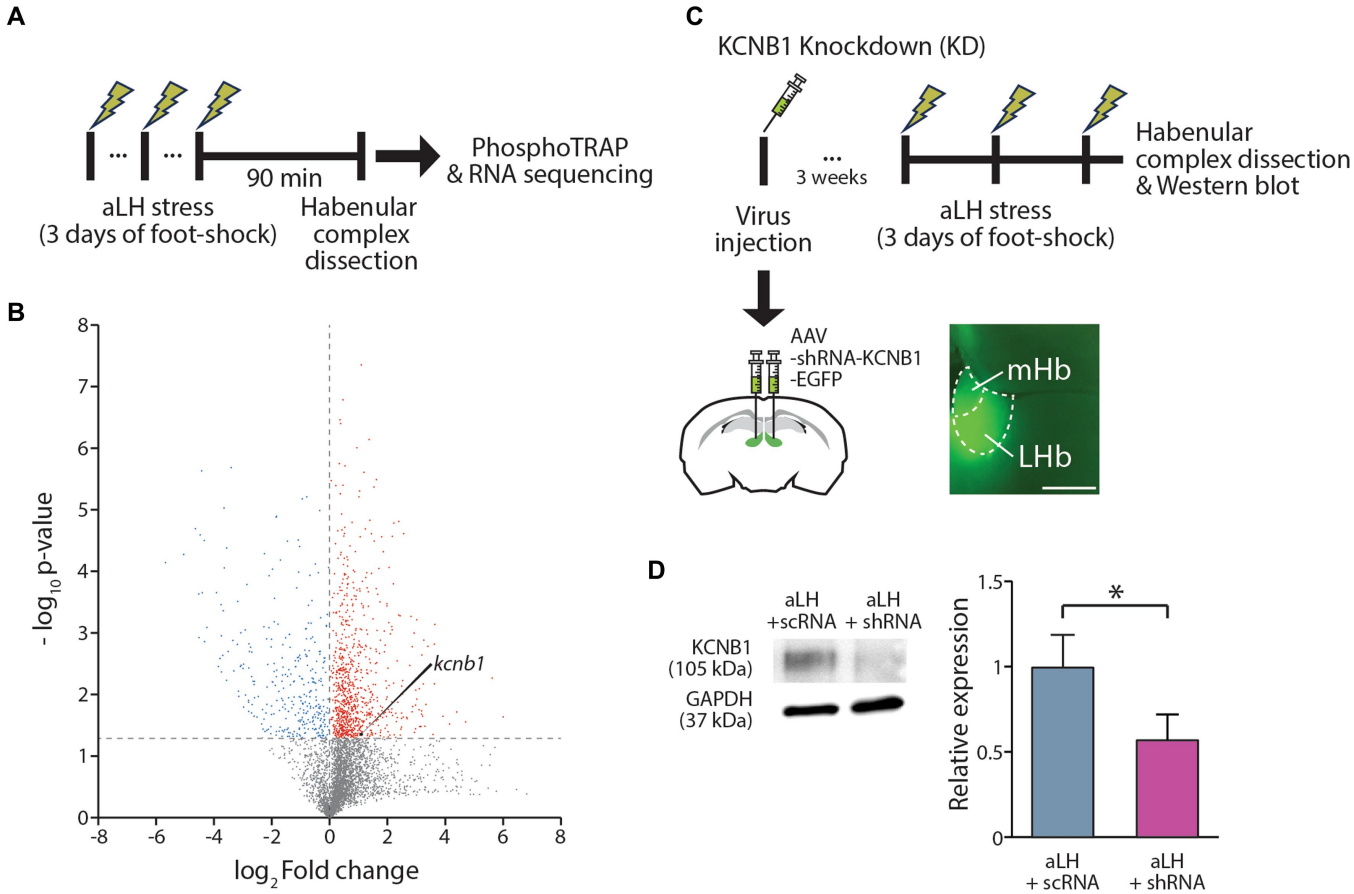
Expression Regulation of KCNB1 in the LHb Under Stress. **(A)** Procedure for the Phospho-TRAP experiments. **(B)** Scatter plot graph depicting gene expression changes. Red dots represent genes were significantly up-regulated after stress exposure, while blue dots indicate down-regulated genes. (C) Procedure for the KCNB1 knockdown at the habenula complex. To block KCNB1 expression, viral infection of shRNA against KCNB1 was conducted at the LHb. (D) Viral delivery of shRNA against KCNB1 reduced the protein levels in the LHb (Student’s t-test, *p* = 0.021).

To obtain a stress-evoked molecular profile selectively from LHb neurons activated by physical stress, mice were exposed to random foot-shocks for 60 min. Ninety minutes after the stress exposure, habenula complex tissues were collected for further analysis (Figure 1A). We collected phosphorylated ribosomes, which are actively translating proteins, and disassociated mRNAs from these ribosomes for RT-PCR. Quantitative comparison of the active mRNA profiles between control and stressed habenula complex revealed approximately 16,000 genes with differential expression (Figure 1B; Supplementary Table S1). Based on fold changes induced by stress and the basal expression level, we selected several candidate genes of interest (Supplementary Table S1). Notably, the expression of *kcnb1*, which encodes the pore-forming and voltage-sensing α subunit of a delayed rectifying voltage-gated potassium channel (KCNB1), was increased by 2 folds during stress (Figure 1B). Considering that fluoxetine treatment inhibits both the expression and activity of the KCNB1 (Chen et al., 2015; Wang et al., 2020), we further investigated the role of KCNB1 in the LHb during the stress exposure.

### KCNB1 reduction alters firing patterns of LHb neurons

**Figure 2:**
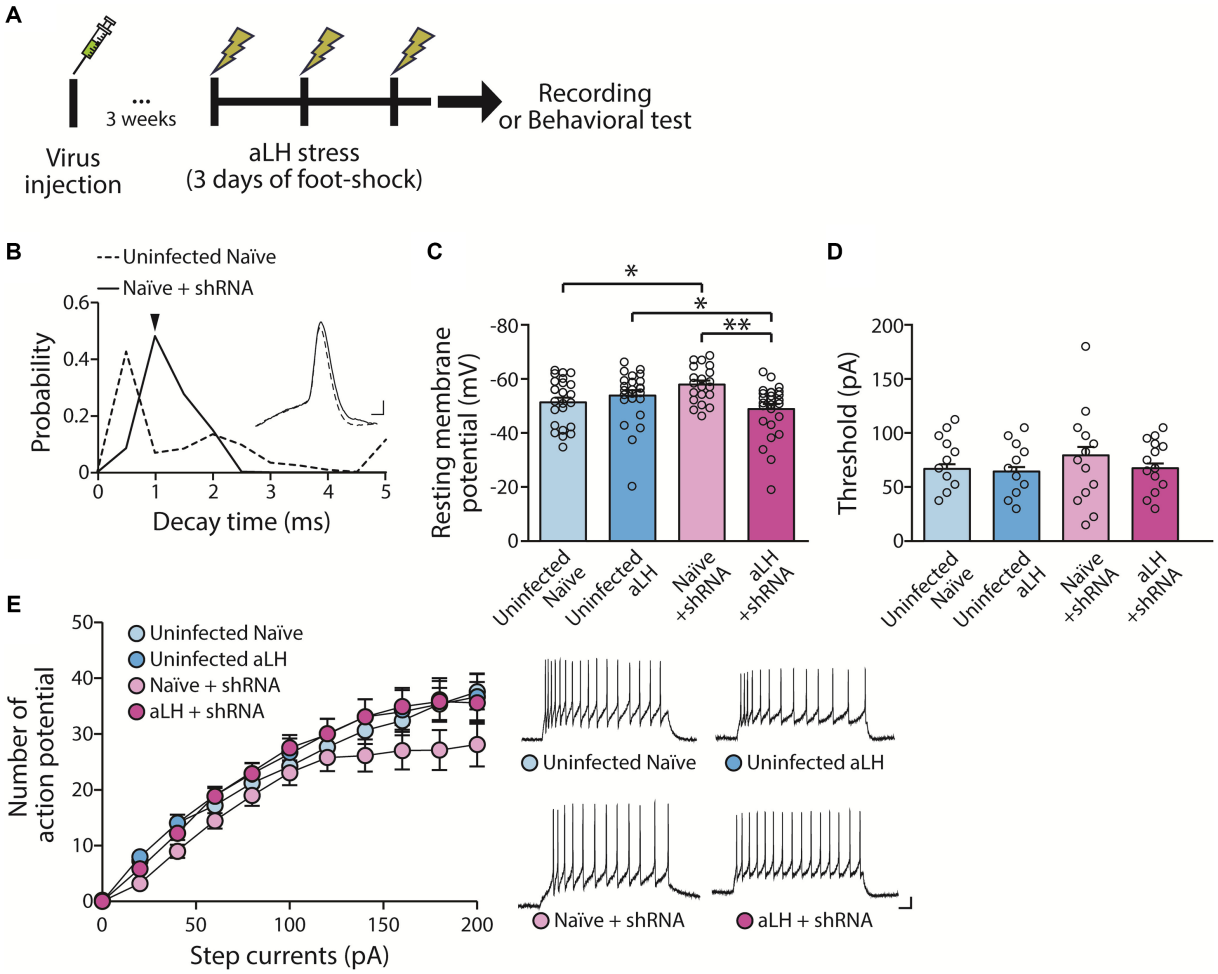
Impact of KCNB1 knockdown on basal neuronal properties. **(A)** Experiment schematic. **(B)** Knockdown of KCNB1 extends the decay time of single action potentials in the LHb (Student’s *t*-test, *p* = 0.016). Representative traces of single action potentials are shown (scale bar: 1 ms, 10 mV). **(C)** Resting membrane potential (RMP) of LHb neurons remains unchanged by acute learned helplessness (aLH) (*n* = 20–27, Kruskal-Walis test, *p* = 0.183 in comparison between Uninfected naïve and Uninfected aLH). KCNB1 knockdown slightly hyperpolarized RMP in naïve group but depolarized in aLH group (Kruskal-Walis test, *p* = 0.023 in comparison between Uninfected naïve and Naïve + shRNA; *p* = 0.014 in comparison between Uninfected aLH and aLH + shRNA). Acute stress exposure induces depolarization of LHb neurons in KCNB1 knockdown groups (Kruskal-Walis test, *p* = 0.001 in comparison between Naïve + shRNA and aLH + shRNA). **(D,E)** Threshold and excitability of neurons show no changes in response to step currents (Two-way ANOVA, main effects of gene modulation, F_1, 97_ = 1.930, *p* = 0.168; main effects of stress exposure, F_1, 97_ = 2.260, *p* = 0.136; interaction, F_1, 97_ = 0.842, *p* = 0.361). Representative traces of excitability at 100 pA current injection for 500 ms are shown (scale bar: 50 ms, 10 mV).

**Figure 3:**
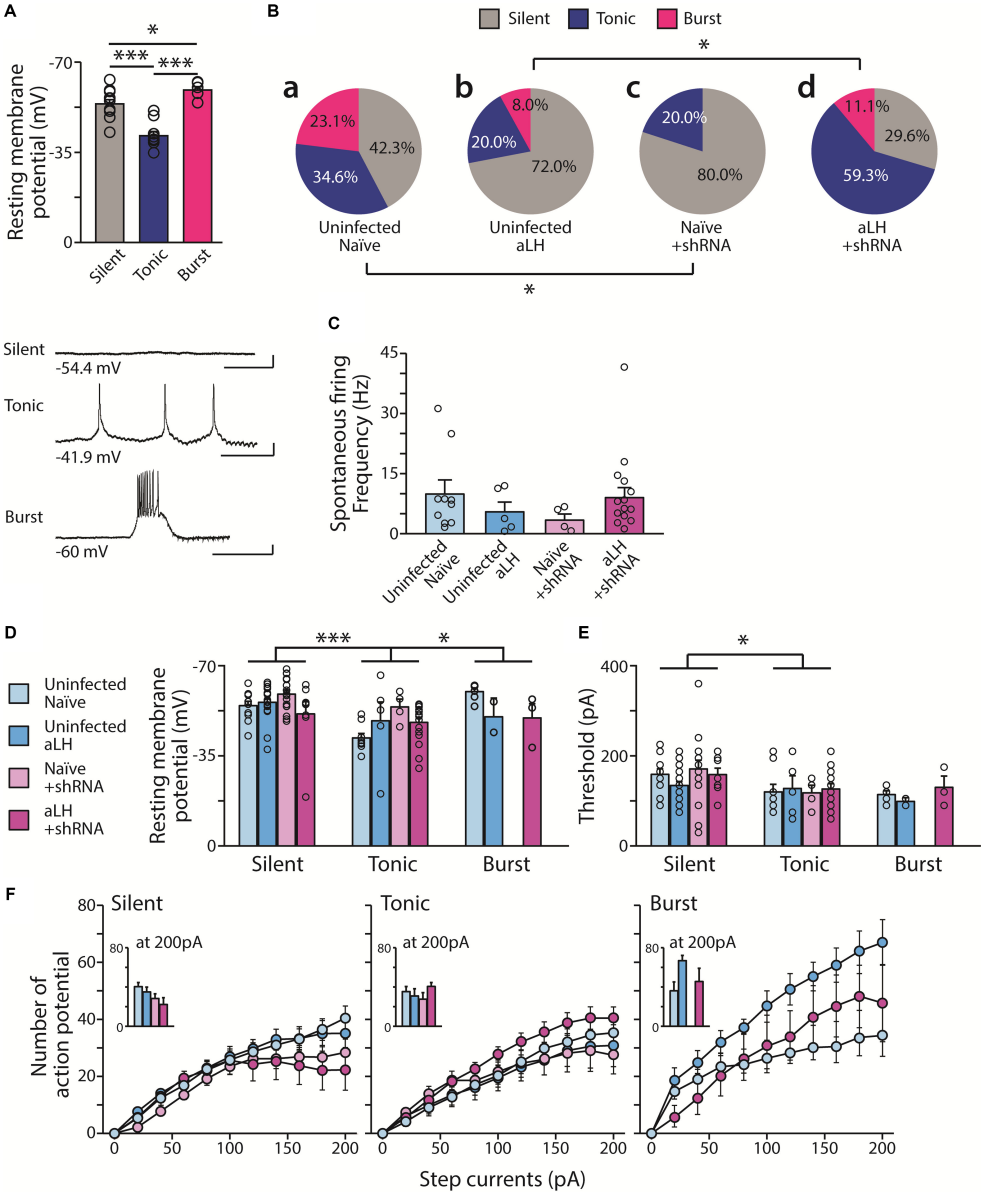
Distribution of firing types with and without acute stress. **(A)** Tonic firing LHb neurons exhibit more depolarized RMP than silent neurons. Burst firing LHb neurons have more hyperpolarized RMP (*n* = 6–11, One-way ANOVA test with LSD post-hoc analysis, F_1,24_ = 26.510, *p* < 0.001; LSD post-hoc analysis, *p* < 0.001 in comparison between Silent and Tonic, *p* = 0.043 in comparison between Tonic and Burst, *p* < 0.001 in comparison between Silent and Burst). Bottom panels show representative traces of firing for each type RMP (scale bar: 400 ms, 10 mV). **(Ba,c)** KCNB1 knockdown increases silent neurons in the naïve group (from 11 out of 26 neurons to 16 out of 20 neurons Pearson chi square test; *p* = 0.017). **(Bb,d)** However, in aLH group, KCNB1 knockdown enhance the relative distribution of firing types (from 18 out of 25 neurons to 8 out of 27 neurons, Pearson chi square; *p* = 0.008). **(C)** Spontaneous firing frequency of tonic firing pattern neurons. There is no effects on spontaneous firing rate with/without both acute stress and KCNB1 expression (*n* = 2–18, Kruskal-Walis test, *p* = 0.496) **(D–F)** Basal neural properties of the three types of LHb neuron (*n* = 0–18). **(D)** RMP of LHb neurons significantly differ depending on stress (F_1,97_ = 3.979, *p* = 0.049) and firing patterns (F_1,97_ = 7.391, *p* = 0.001, two-way ANOVA; LSD *post hoc* analysis, Silent *vs* Tonic; *p* < 0.001, Tonic *vs* Burst; *p* = 0.004) with a significant interaction between KCNB1 knockdown and stress (F_1,97_ = 7.604, *p* = 0.007, Two-way ANOVA) **(E)** Threshold is affected only by the firing patterns (F_1,97_ = 4.356, *p* = 0.016, Two-way ANOVA; LSD *post hoc* analysis, Silent *vs* Tonic; *p* = 0.008, Silent *vs* Burst; *p* = 0.016) **(F)** Excitability of LHb neuron remained unaffected by any factors. Number of action potentials on the series of step currents injection (0 pA to 200 pA for 500 ms duration, 20 pA increment per each step) is plotted. (Insert: Number of action potentials at 200 pA current injection).

To investigate the role of KCNB1 in the LHb during stress, we performed *ex vivo* electrophysiological recordings and behavior tests 3 weeks after the viral delivery of shRNA against KCNB1. The injection of designed virus targeting *kcnb1* mRNA successfully reduced KCNB1 expression to approximately 33% of that observed in the aLH model (Figures 1C,D, *p* = 0.021). Using whole-cell patch clamp recordings, we examined the passive and active properties of LHb neurons with KCNB1 reduction (Figure 2A). Consistent with a previous study (Liu and Bean, 2014), we observed that LHb neurons expressing shRNA exhibited an extended decay time of single action potentials (Figure 2B, *p* = 0.016) as well as hyperpolarized resting membrane potentials (Figure 2C, in *Naïve group*, *p* = 0.023). Interestingly, stress exposure selectively depolarized the resting membrane potentials only in shRNA-infected neurons but not in uninfected aLH (Figure 2C, *p* = 0.001). There were no significant changes in threshold to fire an action potential between groups (Figure 2D, main effects of gene modulation, *p* = 0.168; main effects of stress exposure, *p* = 0.136) or excitability of LHb neurons upon stimulation (Figure 2E, main effects of gene modulation, *p* = 0.389; main effects of stress exposure, *p* = 0.169), suggesting a minimal involvement of KCNB1 in the active properties of LHb neurons. LHb neurons are known to exhibit three different firing patterns: silent, tonic firing, and burst firing (Weiss and Veh, 2011). Our observations of KCNB1 regulating action potential decay time and resting membrane potentials lead to hypothesize that KCNB1 may modulate firing patterns of LHb neurons. To test this hypothesis, we analyzed firing patterns under resting states (I = 0). Consistent with previous studies, we observed three populations of LHb neurons with distinct firing patterns and different resting membrane potentials (Weiss and Veh, 2011) (Figures 3A,B). When we quantified the ratio of each type among total recorded neurons, we found 3 days of random foot-shock stress increased the percentage of silent neurons (from 11 out of 26 neurons to 18 out of 25 neurons) while decreasing the relative percentages of tonic and bursting neurons but no significant (Figures 3Ba,b). Interestingly, reduction of KCNB1 lead to significant decrease in chance to observe burst firing neurons (from 6 out of 26 neurons to 0 out of 20 neurons), resulting in more frequent observations of silent neurons (Figures 3Ba,c, from 11 out of 26 neurons to 16 out of 20 neurons, Pearson chi square test; *p* = 0.017). On the other hand, when KCNB1 expression is limited in aLH mice, the chance for tonic firing was increased whereas the relative percentage of silent neurons decreased compared to non-injected stressed animals (Figures 3Bb,d, from 18 out of 25 neurons to 8 out of 27 neurons, Pearson chi square; *p* = 0.008). These observations suggest that reduced KCNB1 affects firing patterns of LHb neurons and its expression during stress may reshape the overall firing patterns of the LHb. However, there is no significant effect on spontaneous firing frequency regardless of stress exposure or KCNB1 expression (Figure 3C, *p* = 0.496).

KCNB1 is one of most abundant delayed rectifying potassium channels in major brain areas and well known to impact intrinsic excitability of neurons (Du et al., 2000; Malin and Nerbonne, 2002; Liu and Bean, 2014). Therefore, we measured the resting membrane potentials and the threshold to fire an action potential. Interestingly, we found that RMPs differ depending on firing patterns (Figure 3A, *p* = 0.001). The tonic firing neurons exhibited most depolarized RMPs whereas the bursting neurons had relatively hyperpolarized RMPs (Figure 3A, *p* = 0.046). However, there is limited contribution of either the exposure to acute stress or the levels of KCNB1 in RMP (Figure 3D). Threshold to trigger an action potential was measured by injecting brief current pulses (2 ms) and it differs between silent and tonic firing LHb neurons (*p* = 0.016) but not much contribution of stress or KCNB1 knockdown was observed (Figure 3E). To search for additional differences besides the threshold to fire, we measured the excitability of LHb neurons depending on firing types. Injecting a series of step currents (0 pA to 200 pA, 20 pA per each step) at current clamp configuration revealed that burst firing neurons in aLH groups show more higher excitability than other groups of burst firing neuron, but not significant, however the levels of KCNB1 seem to play a minimal role in excitability upon depolarization (Figure 3F).

### Knockdown of KCNB1 reverses enhanced synaptic potentiation of LHb neurons after stress exposure

**Figure 4:**
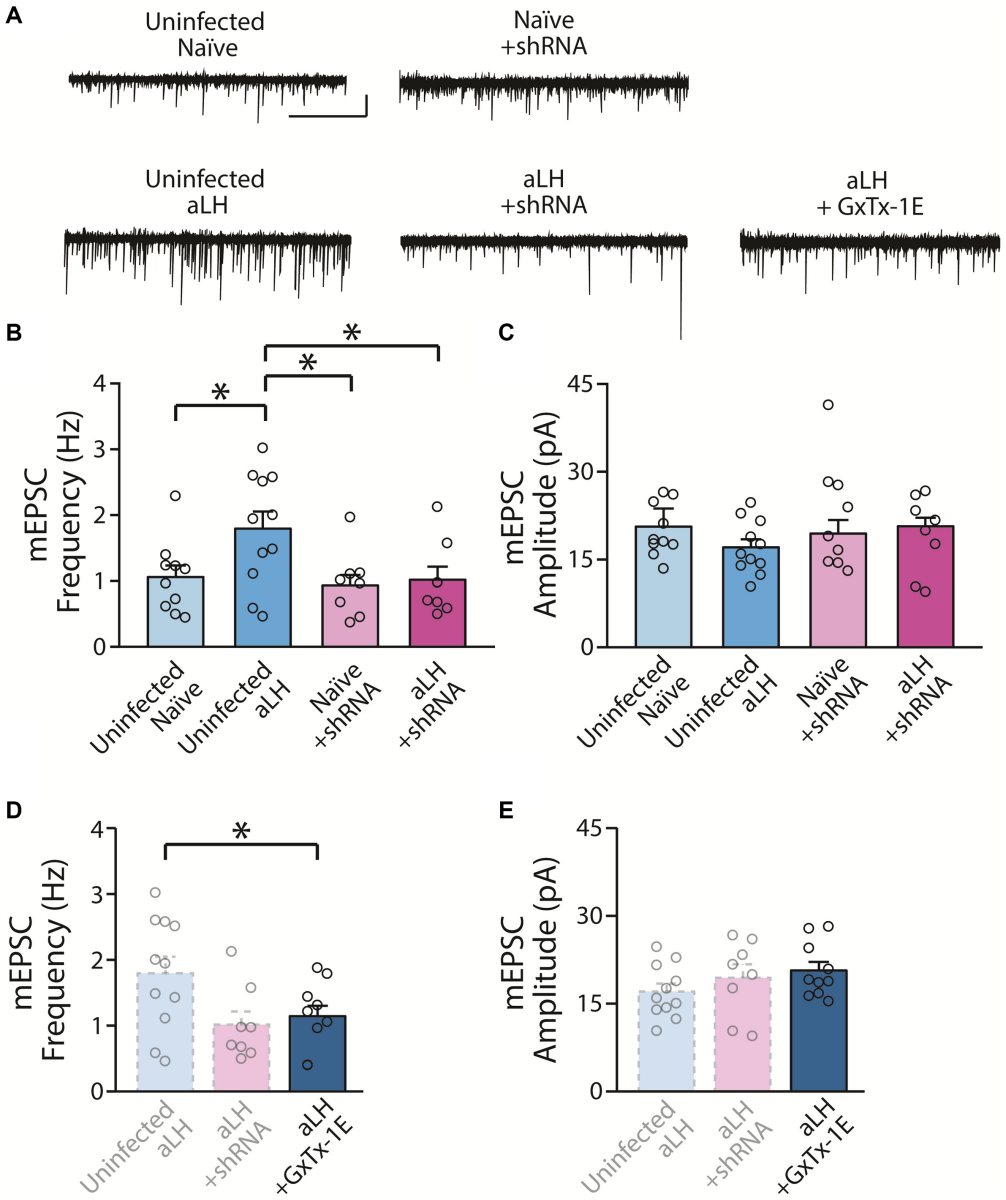
Impact of KCNB1 knockdown on Synaptic Transmission. **(A)** Representative traces of mEPSCs (scale bar: 5 s and 20 pA, n = 7–11). **(B)** KCNB1 knockdown restored the enhancement of mEPSC frequency in the LHb (Two-way ANOVA with LSD post-hoc analysis, main effects of gene modulation, F_1, 37_ = 4.675, *p* = 0.038; main effects of stress exposure, F_1, 37_ = 3.831, *p* = 0.059; interaction, F_1, 37_ = 2.391, *p* = 0.131; LSD post-hoc analysis, *p* = 0.013 in comparison between Uninfected naïve and Uninfected aLH; *p* = 0.013 in comparison between Uninfected aLH and aLH + shRNA). **(C)** No significant changes in mEPSCs amplitudes were observed (Two-way ANOVA with LSD post-hoc analysis, main effects of gene modulation, F_1, 37_ = 0.513, *p* = 0.479; main effects of stress exposure, F_1, 37_ = 0.981, *p* = 0.329; interaction, F_1, 37_ = 0.168, *p* = 0.684). **(D,E)** Pharmacological blockade of KCNB1 with GxTx-1E (KCNB1 antagonist) application. **(D)** Blocking of KCNB1 function with GxTX-1E reversed the increased of mEPSCs frequency after acute stress exposure (One-way ANOVA test with LSD post-hoc analysis, F_2, 28_ = 3.867, *p* = 0.034; LSD post-hoc analysis, *p* = 0.019 in comparison between Uninfected aLH and aLH + shRNA; *p* = 0.036 in comparison between Uninfected aLH and aLH + GxTx-1E) **(E)** but not amplitude (One-way ANOVA test, F_2, 28_ = 1.292, *p* = 0.292).

To examine the action of KCNB1 on synaptic transmission in the LHb, we recorded spontaneous synaptic transmission under voltage-clamp conditions at - 60 mV. Consistent with a previous study, we observed a significant increase in mEPSCs frequency in aLH group (Li et al., 2011). Viral infection of scrambled RNA (scRNA) had no effects on basal synaptic transmission with or without acute stress exposure (Supplementary Figure, in comparison of *uninfected* and *scRNA* group, *p* = 0.503 for naïve; *p* = 0.864 for aLH). We observed that mEPSC frequency of LHb neurons was increased in aLH mice (Figures 4A,B, in comparison of *uninfected naïve* and *uninfected aLH* groups, *p* = 0.013). Interestingly, knockdown of KCNB1 successfully blocked facilitation of mEPSC frequency of the LHb after stress exposure (Figures 4A,B, in comparison of *Naïve + shRNA* and *aLH + shRNA*, *p* = 0.784) without affecting mEPSCs amplitudes (Figures 4A,C, main effects of gene modulation, *p* = 0.479; main effects of stress exposure, *p* = 0.329). Bath application of a specific KCNB1 blocker, GxTx-1E (Frazzini et al., 2016) also inhibit the stress-induced increase in mEPSCs frequency in the LHb (Figures 4A,D, *p* = 0.036) but no effects on amplitude (Figures 4A,E, *p* = 0.292). These observations suggest that increased KCNB1 after stress exposure contributes to the synaptic enhancement induced by stress exposure.

**Figure 5:**
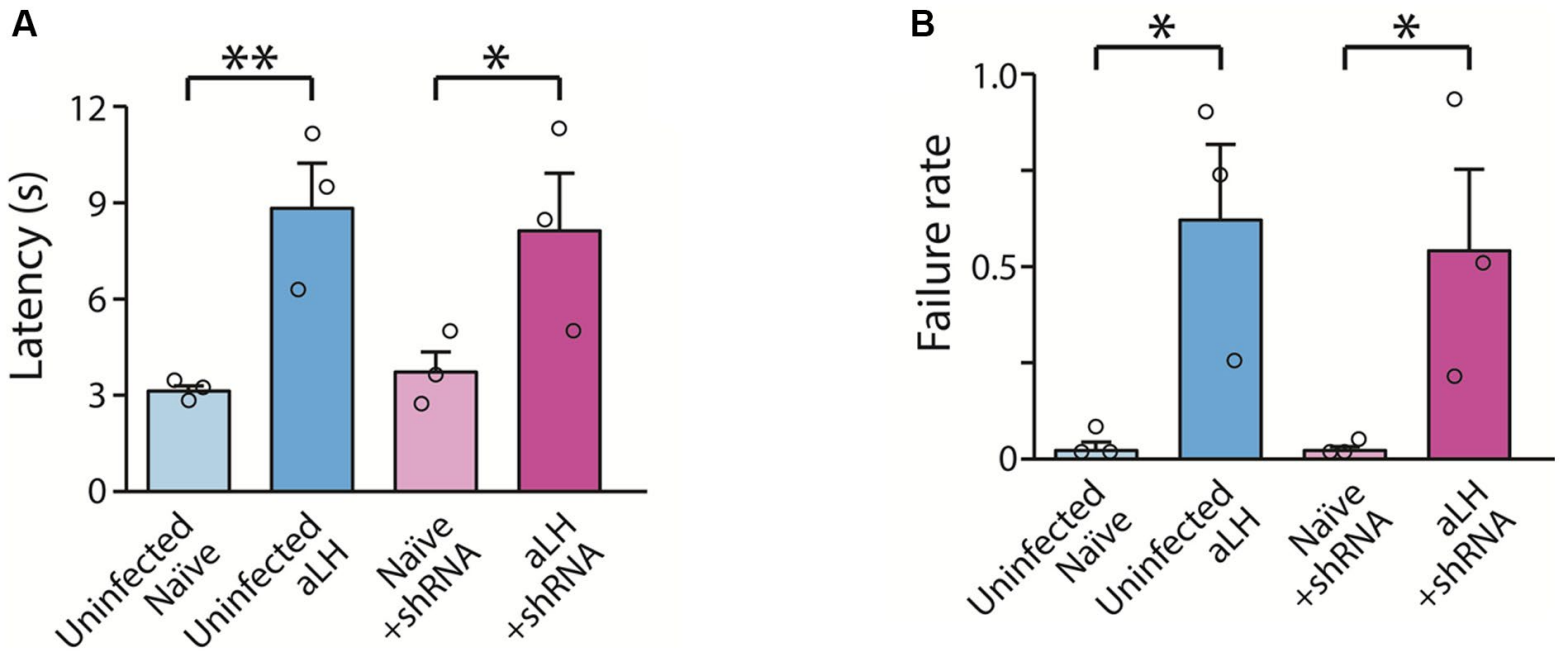
The impacts of KCNB1 on despair behaviors. **(A,B)** KCNB1 knockdown does not restore despair behaviors of aLH animals. **(A)** After stress exposure, escape latency was increased (Two-way ANOVA with LSD post-hoc analysis, main effect of stress, F_1,11_ = 18.111, *p* = 0.003; LSD post-hoc analysis, *p* = 0.009 in comparison between Uninfected naïve and Uninfected aLH; *p* = 0.031 in comparison between Naïve + shRNA and aLH + shRNA), but there was no effect of KCNB1 knockdown (Two-way ANOVA, main effect of gene modulation, F_1,11_ = 0.002, *p* = 0.967). Failure rate was increased after stress exposure as well as KCNB1 knockdown groups (Two-way ANOVA with LSD post-hoc analysis, main effect of stress, F_1,11_ = 14.636, *p* = 0.005; LSD post-hoc analysis, *p* = 0.019 in comparison between Uninfected naïve and Uninfected aLH; *p* = 0.035 in comparison between Naïve + shRNA and aLH + shRNA), but there was no effect of KCNB1 knockdown (Two-way ANOVA, main effect of gene modulation, F_1,11_ = 0.07, *p* = 0.798). There was no interaction between stress and gene modulation effect (Two-way ANOVA, interaction, F_1,11_ = 1.316, *p* = 0.598 for latency; F_1,11_ = 0.005, *p* = 0.798 for failure rate).

Next, to investigate whether the increased KCNB1 contributes to depressive-like behavior or not, we bilaterally delivered AAV-shRNA against KCNB1 into the LHb and tested the animals in the active avoidance test. Acute foot-shock stress increased the escape latency as compared to those of uninfected and shRNA injected animals as reported previously (Banasr and Duman, 2008) (Figures 5A,B, in comparison of *uninfected naïve* and *aLH*, *p* = 0.009; in comparison of *Naïve + shRNA* and *aLH + shRNA*, *p* = 0.031). Furthermore, we observed increased failure rates in aLH group, however no significant improvement by KCNB1 knockdown was observed (Figures 5A,B, in comparison of *uninfected naïve* and *aLH*, *p* = 0.019; in comparison of *Naïve + shRNA* and *aLH + shRNA*, *p* = 0.035). Our data indicate that KCNB1 knockdown in an aLH animal model did not show any improvement in escape time, or a failure rate compared to the aLH group, suggesting that the selective knockdown of KCNB1 may not be enough to ameliorate active coping behaviors observed in aLH animals.

## Discussion

In this study, we obtained a molecular profiling of the LHb during acute physical stress and examined the roles of *kcnb1*, one of stress-induced activated genes in the LHb. It is now very well established that the enhanced activity of the LHb is critical in patients and animal models of depression (Li et al., 2011, 2013; Park et al., 2017b; Seo et al., 2018). Several studies reveled candidate molecules that can increase synaptic activity of the LHb and induce depressive-like behaviors (Li et al., 2013; Cui et al., 2018; Seo et al., 2018). However, molecular changes occurring during stress exposure had not been investigated. Among a list of genes we identified, we chose *kcnb1* for further study based on its possible involvement in stress responses (Chen et al., 2015; Wang et al., 2020) and anticipated roles in regulating synaptic activity of the LHb (Li et al., 2011, 2013; Park et al., 2017b; Seo et al., 2018).

We observed that knockdown of KCNB1 hyperpolarized resting membrane potentials and alter the ratio of firing pattern (silent, tonic firing, burst firing) distribution of LHb neurons. KCNB1 is known to be located at symmetrical synapses facing astrocytes (Du et al., 1998). It is known that extracellular K^+^ concentration is mainly determined by actions of potassium channels and astrocytes can indirectly participate neurotransmitter release through depolarization of presynaptic neurons (Stuart et al., 1997).

Recent studies suggest that changes in K^+^ concentration can alter firing patterns of LHb neurons (Cui et al., 2018; Yang et al., 2018a). In animal models of depression, increased K^+^ currents through Kv4.1 in astrocytes within the LHb led to a decrease in K^+^ concentration around neurons. A decrease in extracellular K^+^ concentration hyperpolarizes the membrane potential, leading to the activation of T-type calcium channels and the induction of bursting firings (Cui et al., 2018; Yang et al., 2018a). In fact, LHb neurons are reported to exhibit increased burst firing following chronic stress (Yang et al., 2018a). However, our study found a greater proportion of silent neurons compared to burst firing neurons after acute stress exposure (Figure 3Bb). This inconsistency may be originated from the duration of stress as reported previously (Zheng et al., 2022). Compared the previous study, the locations of target channels (neurons versus astrocytes) as well as the equilibrium potential of potassium also differ. Notably, individual LHb neurons exhibit varied firing patterns depending on their membrane potentials (Weiss and Veh, 2011). Therefore, our observations may have been influenced by the internal and external K^+^ concentration used in the experiments. Specifically, calculated equilibrium potentials for potassium (E_K_) differ between a previous study and ours: In our recording conditions, E_K_ is estimated around −98 mV while it was around −104 mV in the previous study (Yang et al., 2018a). The difference in E_K_ can affect the theoretical electrochemical driving force for K^+^ at the holding potential. Additionally, a previous study reported that reducing extracellular K^+^ concentration gradually hyperpolarizes RMP and alters firing pattern of LHb neuron from tonic firing to burst firing (Cui et al., 2018). They also observed that artificially injected current alters neuronal RMP and firing patterns. Intentional hyperpolarization by injecting negative current (− 20 pA) induced burst firing from tonic firing neurons, whereas depolarization by positive current injection (+ 20 pA) lead to tonic firing from burst firing neurons (Yang et al., 2018a).

In our analysis of firing patterns, the control KCNB1 knockdown group showed an absence of burst firing LHb neurons (Figure 3Bc). Additionally, KCNB1 knockdown in the aLH group displayed an increased population of tonic firing neurons. Changes in firing patterns in both groups may be regulated by the activity-dependent function of KCNB1. A previous study in the hippocampus revealed that KCNB1 inhibition suppresses repolarization of action potentials, leading to prolonged depolarization and increased spontaneous firing (Liu and Bean, 2014). Interestingly, opposite effects were noted in bursting neurons. Continuous current injection induced initial action potentials effectively, but later induced action potentials were not well sustained with a KCNB1 blocker, resulting in reduced bursting. Inhibition or reduced expression of KCNB1 thus impedes repolarization of action potentials, thereby increasing tonic activity and disrupting bursting.

Despite being an ion channel, we observed that knockdown of KCNB1 regulates spontaneous synaptic transmission in the LHb (Figure 4). This observation is interest considering the enhanced synaptic transmission onto LHb neurons in depression animal models (Li et al., 2011). KCNB1 knockdown resulted in reduced mEPSC frequency of LHb neurons in the aLH group and so did the pharmacological blockade of KCNB1. KCNB1 has been reported to interact with voltage-gated calcium channels at the axon terminal and endoplasmic reticulum (ER), regulating synaptic transmission in the presynaptic side (Frazzini et al., 2016). Conditional knockdown of KCNB1 using shRNA inhibits calcium influx into the axon terminal, reducing presynaptic vesicle release. However, the postsynaptic mechanisms of KCNB1 in synaptic transmission remain unknown. Given the coupling of KCNB1 with postsynaptic L-type calcium channels (LTCC) or ryanodine receptors in the ER, KCNB1 may impact the levels of intracellular calcium, which plays a pivotal role in synaptic transmission (Vierra et al., 2019). It is well known that LTCC leads to calcium influx into the neurons and affects synaptic potentiation (Da Silva et al., 2013; Wiera et al., 2017). LTCC is clustered by KCNB1 (Vierra et al., 2019), therefore, increased KCNB1 expression during stress exposure could enhance calcium influx and induce plastic changes in synapses through downstream LTCC signaling. Further research may provide a better delineation for the relationship between KCNB1 and synaptic transmission.

Animal models of depression show increased activity in the LHb both in terms of synaptic transmission and active firing. The correlation between enhanced presynaptic inputs, firing rates, and burst firing with depression-related behaviors is well established (Li et al., 2011, 2013; Cui et al., 2018; Yang et al., 2018a). However, KCNB1 knockdown did not alleviate despair behaviors in our study. Although KCNB1 knockdown resulted in decreased mEPSCs, the changes in firing patterns and excitability were not restored to control group levels, potentially hindering the restoration of depressive behaviors. A molecule implicated in LHb hyperactivation in an animal model of depression, referred to as p11, successfully restored mEPSCs as well as burst firing patterns in a chronic stress model, contributing to the recovery of depressive behaviors in animals (Seo et al., 2018). These findings suggest that simultaneous restoration of both synaptic activity (such as miniature transmission) and active neuronal properties (such as bursting) matter to induce antidepressant effects at the level of behaviors in depressed animals. While KCNB1 knockdown did not yield behavioral improvement, this observation does not diminish the role of KCNB1 in stress response. Rather, these observations imply that blocking stress-induced KCNB1 translation might not be sufficient to evoke depression-like behaviors. However, the rapid induction of KCNB1 in response to acute stressors participate in the synaptic enhancement of the LHb, which is critical for behavioral deficits observed in depression animal models as well as patients (Sartorius et al., 2010; Li et al., 2011; Yang et al., 2018b).

One thing to consider in our study is that the MHb also expresses KCNB1 (Mandikian et al., 2014) and it is possibility that acute stress may impact on KCNB1 expression in the MHb. We used the habenular complex containing both lateral and medial parts to ensure the quantity of RNAs enough for phospho-TRAP experiments. While we cannot entirely rule out post-stress changes in the MHb, we expect that the molecular changes in the MHb during the stress might not be substantial. Previous studies have reported that the MHb is not so responsive to stress (Park et al., 2017b; Kim and Chung, 2021). Expression of c-fos upon stress was selectively observed in the LHb, but not in the MHbs (Park et al., 2017b). In addition, activated neurons were reported to concurrently express c-fos and phosphorylated S6 in ribosomes (Knight et al., 2012). We revealed that stress-induced changes in the LHb can be restored by blocking KCNB1 function with GxTx-1E (Figure 4), providing further support for the importance of KCNB1 in the LHb.

A limitation of our study is that shRNA delivery for expression manipulation may not be adequate to induce behavioral amelioration. Our manipulation using shRNA also lacks the timing of suppression, which may be critical to induce such behavioral changes. An increase in KCNB1 expression was observed both during and after stress, and suppression of KCNB1 expression led to recovery from abnormal synaptic hyperactivity in addition to altering firing patterns of LHb neurons in an animal model of depression. However, it did not significantly change the overall activity of the LHb, possibly due to technical limitations of shRNA-induced reduction. Achieving a more precise manipulation of KCNB1 activity, along with identifying cellular and molecular signals in response to changes in KCNB1 expression, would be of great interest. The gene sets obtained through our phospho-TRAP experiments will offer valuable targets for future research, suggesting substances that can modulate LHb activity in conjunction with KCNB1.

## Data availability statement

The original contributions presented in the study are publicly available. This data can be found here: GSE249051.

## Ethics statement

The animal study was approved by Institutional Animal Care and Use Committee (IACUC) of Konkuk University (KU22005, Seoul, South Korea) and IACUC of Daegu Gyeongbuk Institute of Science and Technology (DGIST, Daegu, South Korea). The study was conducted in accordance with the local legislation and institutional requirements.

## Author contributions

HR: Data curation, Formal analysis, Investigation, Writing – review & editing. MK: Methodology, Writing – review & editing. HP: Data curation, Formal analysis, Visualization, Writing – original draft, Writing – review & editing. HC: Methodology, Writing – review & editing. CC: Conceptualization, Data curation, Formal analysis, Funding acquisition, Investigation, Project administration, Supervision, Validation, Writing – original draft, Writing – review & editing.
