# Supplementary material for: Stress-induced translation of KCNB1 contributes to the enhanced synaptic transmission of the lateral habenula
Source: Front Cell Neurosci. 2023 Dec 13;17:1278847. doi: 10.3389/fncel.2023.1278847 (PMC10773861; doi:10.3389/fncel.2023.1278847)
Supplement: Supplementary file 1 [file Data_Sheet_1.docx]

Supplementary figure

KCNB1 regulates synaptic transmission in the lateral habenula during stress exposure.

Hakyun Ryu^1^, Minseok Kim^2^, Hoyong Park^1^, Han Kyoung Choi^2^, and ChiHye Chung^1, *^

^1^Department of Biological Sciences, Konkuk University, Seoul 05029, Republic of Korea

^2^Department of Brain and Cognitive Sciences, DGIST, Daegu 42988, Republic of Korea

*** Correspondence: ChiHye Chung, Ph.D.,** [cchung@konkuk.ac.kr](mailto:jshan06@konkuk.ac.kr)


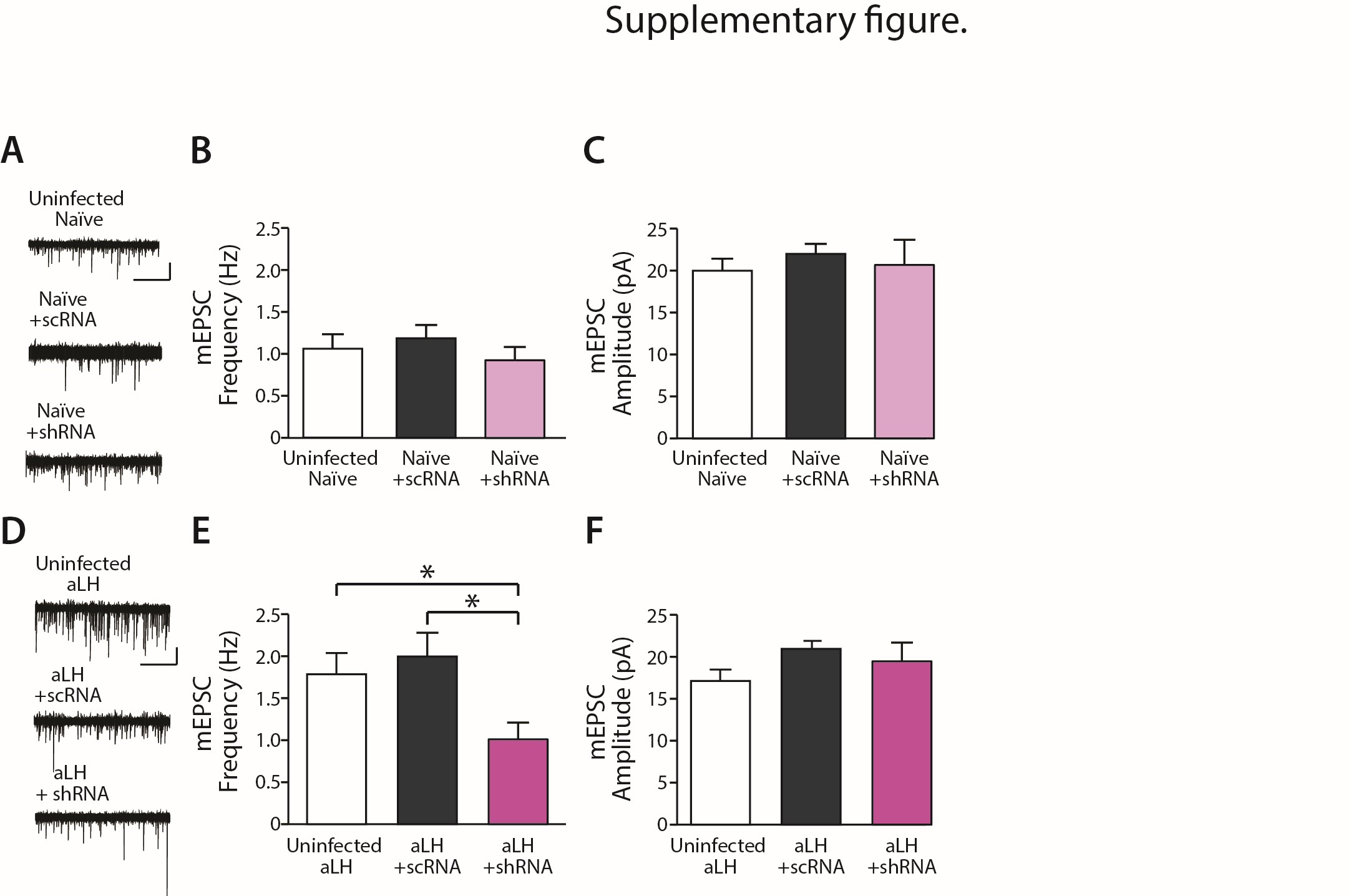


**Supplementary Figure 1.** Basal synaptic transmission of the LHb neurons between uninfected and viral infected groups. (A) Representative trace of mEPSCs of the Naïve groups (B and C) In unstressed animals, there were no significant alterations in mEPSC frequency and amplitude (n = 9-22, Kruskal-Walis test, p = 0.503 for frequency; p = 0.374 for amplitude). (D) Representative mEPSCs trace of aLH groups. (E) In aLH animal model, there was no difference in mEPSC frequency between uninfected aLH and aLH + scRNA group. However, KCNB1 knockdown restored enhancement of mEPSCs frequency after stress exposure (n = 8-23, Kruskal-Walis test, p = 0.864 in comparison between Uninfected aLH and aLH + scRNA; p = 0.030 in comparison between Uninfected aLH and aLH + shRNA; p = 0.022 in comparison between aLH + scRNA and aLH + shRNA) (F) There were no differences in amplitude (Kruskal-Walis test, p = 0.163).
